# Intracellular Progesterone Receptor and cSrc Protein Working Together to Regulate the Activity of Proteins Involved in Migration and Invasion of Human Glioblastoma Cells

**DOI:** 10.3389/fendo.2021.640298

**Published:** 2021-03-26

**Authors:** Claudia Bello-Alvarez, Aylin Del Moral-Morales, Aliesha González-Arenas, Ignacio Camacho-Arroyo

**Affiliations:** ^1^ Unidad de Investigación en Reproducción Humana, Instituto Nacional de Perinatología-Facultad de Química, Universidad Nacional Autónoma de México (UNAM), Ciudad de México, Mexico; ^2^ Departamento de Medicina Genómica y Toxicología Ambiental, Instituto de Investigaciones Biomédicas, UNAM, Ciudad Universitaria, Ciudad de México, Mexico

**Keywords:** glioblastoma, progesterone receptor, cSrc, non-genomic actions, focal adhesion kinase, paxillin

## Abstract

Glioblastomas are the most common and aggressive primary brain tumors in adults, and patients with glioblastoma have a median survival of 15 months. Some alternative therapies, such as Src family kinase inhibitors, have failed presumably because other signaling pathways compensate for their effects. In the last ten years, it has been proven that sex hormones such as progesterone (P4) can induce growth, migration, and invasion of glioblastoma cells through its intracellular progesterone receptor (PR), which is mostly known for its role as a transcription factor, but it can also induce non-genomic actions. These non-classic actions are, in part, a consequence of its interaction with cSrc, which plays a significant role in the progression of glioblastomas. We studied the relation between PR and cSrc, and its effects in human glioblastoma cells. Our results showed that P4 and R5020 (specific PR agonist) activated cSrc protein since both progestins increased the p-cSrc (Y416)/cSrc ratio in U251 and U87 human glioblastoma derived cell lines. When siRNA against the PR gene was used, the activation of cSrc by P4 was abolished. The co-immunoprecipitation assay showed that cSrc and PR interact in U251 cells. P4 treatment also promoted the increase in the p-Fak (Y397) (Y576/577)/Fak and the decrease in p-Paxillin (Y118)/Paxillin ratio, which are significant components of the focal adhesion complex and essential for migration and invasion processes. A siRNA against cSrc gene blocked the increase in the p-Fak (Y576/Y577)/Fak ratio and the migration induced by P4, but not the decrease in p-Paxillin (Y118)/Paxillin ratio. We analyzed the potential role of cSrc over PR phosphorylation in three databases, and one putative tyrosine residue in the amino acid 87 of PR was found. Our results showed that P4 induces the activation of cSrc protein through its PR. The latter and cSrc could interact in a bidirectional mode for regulating the activity of proteins involved in migration and invasion of glioblastomas.

## Introduction

Astrocytomas are the most common primary brain tumors in the central nervous system (CNS). The WHO classifies these tumors according to the degree of malignancy in a range from I to IV. Grade IV represents the most malignant astrocytoma, also known as glioblastoma (1). Patients with glioblastoma have an overall survival of 15 months, even when receiving the standard therapy consisting of the maximum bearable surgical removal followed by radiotherapy and chemotherapy with temozolomide (2). The current standard treatment for glioblastoma has remained unchanged for more than ten years (3). Some alternatives, such as the use of angiogenesis blockers or Src family kinase inhibitors, have been tested in clinical assays, but none of them with successful outcomes (4, 5) The poor prognosis of patients with glioblastoma is a consequence of the high rate of recurrence of these tumors promoted by the inherently radio-resistance and chemo-resistance and the high rate of migration and tumor invasion cells (6). Glioblastoma cells can spread to the surrounding brain parenchyma, which makes extremely difficult the complete resection of the tumor, and finally provokes the recurrence of glioblastoma (7).

Migration and invasion of tumor cells to the normal brain are complex processes that involve multiple steps and molecular signaling. In this context, the focal adhesion complex has a significant role. Some of their structural and regulator components, including non-receptor cytoplasmic tyrosine kinase cSrc, Focal adhesion kinase (Fak), Paxillin (Pax), Tyrosine-protein phosphatase non-receptor type 12 (PTP-PEST), and integrins have been associated with the spread of glioblastoma cells (8–11). The tyrosine kinase Fak acts as a regulator and scaffold protein since it can recruit cSrc and Pax to the specific sites in the focal adhesion complex. In turn, cSrc can phosphorylate other proteins, including Fak and Pax to form an active complex able to mediate the cell-extracellular matrix (ECM) adhesion, protrusion of cytoplasm to form the leading edge, cell contraction, recruitment of proteases, and detachment of the trailing edge (12). The role of PTP-PEST in glioblastomas has been associated with the stability of focal adhesion substrates (Fak, Pax, among others) by the regulation of their phosphorylation-dependent ubiquitination (11).

Lewis-Tuffin and colleagues demonstrated that some Src Family Kinase members, such as cSrc, Fyn, Yes, and Lyn, have an essential role in the motility of glioblastoma cells since the knockdown of these kinases reduces the rate of migration in three different cell lines (8). Some stem cell markers, such as Oct-3/4 have been related to increased migration and invasion of glioma stem cells through cSrc and Fak upregulation (13).

As a result of the higher prevalence of glioblastoma in men than women (14), sex hormones and their receptors have gained particular attention. Several studies have demonstrated a central role of progesterone (P4) in the promotion of proliferation (15, 16), migration, and invasion (17) of glioblastoma cells. One of the proteins with a great affinity for P4 is the progesterone receptor (PR), which belongs to the nuclear receptor family, and acts as a ligand-inducible transcription factor (18). When oligonucleotide antisense against PR or RU486, an antagonist of PR, was administered, the effect of P4 over migration and invasion on human glioblastoma cells was significantly diminished (17). These results suggest that PR has a significant role in promoting the progression of glioblastomas. In some breast cancer cell lines, it has been proven that P4 activates cSrc through PR, and in turn, increases migration and invasion rate (19). However, the role of PR in cSrc activation and their participation in the migration and invasion of glioblastoma cells is unknown. In this work, we studied the interplay between PR and cSrc, and its effects on the activity of proteins involved in migration and invasion of glioblastoma cells. To study the potential relationship between these proteins, glioblastoma-derived cell lines were treated with P4 or R5020 (PR agonist), and the phosphorylated/non-phosphorylated ratio of cSrc was measured by western blot. P4 and R5020 increased cSrc phosphorylation. To confirm the participation of PR in the cSrc phosphorylation, cells were transfected with a commercial siRNA against PR. Cells transfected with the PR siRNA were unable to increase cSrc phosphorylation. To investigate the physical interaction between PR and cSrc, we performed a co-immunoprecipitation assay, and interaction between PR and cSrc was observed. *In silico* analysis showed that cSrc could participate in the phosphorylation of PR in the amino acid 87. The role of cSrc activation by P4 in the switch Fak-phosphofak and Pax-phosphopax ratios and the migratory capacity of glioblastoma cells was determined by western blot and wound-healing assay in cells transfected with a commercial siRNA against cSrc. Fak phosphorylation and migration decreased in cells transfected with siRNA against cSrc compared to cells treated with control siRNA. Findings of this work suggest for the first time that cSrc and PR interact in glioblastoma cells. P4 through PR induces cSrc activation, which in turn participates in regulating the activity of proteins involved in the migration and invasion of glioblastomas.

## Materials And Methods

### Cell Culture and Treatments

U251 and U87 (ATCC, USA) human glioblastoma derived cell lines were plated in 10 cm dishes and sustained in DMEM medium (*In vitro*, S.A., D.F., MEX), supplemented with 10% fetal bovine serum (FBS), 1 mM pyruvate, 2 mM glutamine, 0.1 mM non-essential amino acids (GIBCO, NY, USA) at 37°C, 5% CO2. The culture medium mentioned above was replaced by DMEM medium (*In vitro*, S.A., CDMX., MEX) without phenol red and free of hormones, supplemented with charcoal-stripped serum FBS (sFBS) (Hyclone, Utah), 24 h before the treatments. Cells were treated with P4 (10, 50 and 250 nM), 10 nM of R5020 (progestin with high affinity for PR (Kd ≈ 2 nM)) (20) or vehicle (DMSO 0.001%). Cell treatments lasted 10 and 20 min to assess cSrc, Fak, and Pax phosphorylation.

### Protein Extraction and Western Blotting

Activation of cSrc, Fak, and Pax was determined by measuring protein phosphorylation. Cells were treated with P4 (10, 50, and 250 nM), R5020 (10 nM), or vehicle (DMSO 0.001%), and western blot was used to determine the content of p-cSrc, p-Fak, and p-Pax. After treatments, cells were homogenized in RIPA buffer with a cocktail of protease inhibitors (Sigma Aldrich, St Louis, MO USA, # P8340) and a group of phosphatase inhibitors (NaF, Na_4_P_2_O_7_, and Na_3_VO_4_). Proteins were obtained by centrifugation at 12,500 rpm for 5 min and quantified using the NanoDrop-2000 spectrophotometer (Thermo Scientific, MA, USA). For protein separation, 30 µg were loaded on a polyacrylamide gel at a concentration of 8.5% for cSrc and Pax, and 7.5% for Fak, under denaturing conditions. Proteins were transferred to a nitrocellulose membrane under semi-dry conditions in a transfer (BIO-RAD) for 30 min at 25 V in the case of the 60 kDa (cSrc) and 68 kDa (Pax) proteins and 1 h at 25 V in the case of the 125 kDa protein (Fak). Blocking was performed with 5% bovine serum albumin at 37°C for 2 h. Membranes were incubated with the primary antibodies against the phosphorylated and total forms of the cSrc, Pax, and Fak proteins (phospho Src Tyr-416 Cell Signaling, MA, USA, Ref. 2101; Src Cell Signaling, MA, USA. USA, Ref. 2108; phospho Pax Tyr-118 Cell Signaling, MA, USA, Ref. 2541; Pax Cell Signaling, MA, USA, Ref. 2542; phospho Fak Tyr-397 Cell Signaling, MA, USA, Ref. 3283; Fak Cell Signaling, MA, USA, Ref. 3285). Antibodies against the total and phosphorylated forms were used in a 1/500 dilution. As a loading control, the alpha-tubulin protein was detected at a 1/1,000 dilution (Santa Cruz Biotechnology, St. Louis, TX, USA, Ref. sc-398103). All the antibodies were incubated for 48 h except that against alpha-tubulin, which was incubated for 24 h. Subsequently, the membranes were incubated with the secondary antibody against rabbit (Thermo Scientific, USA, Ref. 1858415) or mouse (Santa Cruz Biotechnology, TX, USA, Ref. sc-516102) (1/10,000) with shaking and at room temperature for 45 min. The primary and secondary antibodies were removed from the membranes with a solution containing Tris–HCl pH 6.8 at 0.06 M, SDS at 2%, and β-mercaptoethanol at 0.7% for 30 min at 50°C at stirring. The chemiluminescent signal was detected by exposing the membranes to the SuperSignal West Fento substrate (Thermo Scientific # 34096) with Kodak Biomax Light Film plates (Sigma-Aldrich, MO, USA).

### siRNA Transfection

Commercial siRNA against PR was used to test if P4 induced the cSrc activation through its PR. Briefly, 2.5×10^5^ U251 cells were plated in 6-well dishes in DMEM medium supplemented with 10% FBS, and 24 h later, the medium was replaced with DMEM phenol red-free medium without FBS and antibiotics. Cells were transfected with a PR siRNA (100 nM) or with control siRNA that does not induce specific mRNA degradation using Lipofectamine RNAiMAX (Thermo Scientific, USA). The medium was refreshed 12 h after the addition of a PR siRNA or control siRNA, and 48 h after siRNAs addition, the cells were harvested for total RNA extraction to determine the efficiency of the transfection. The same protocol was used with Commercial siRNA against cSrc to test the interplay between P4, cSrc, and Fak and Pax activation. In this case, the efficiency of transfection was determined by western blot, and 48 h after transfection, the cells were harvested for protein extraction as previously described.

### RNA Extraction and RT-PCR

RNA extraction was performed using TRIzol reagent (Invitrogen, USA) and following the manufacturer’s instructions. One µg of total RNA was used to synthesize the first-strand cDNA in a reaction carried out by M-MLV reverse transcriptase (Thermo Scientific, USA) following the manufacturer’s protocol. The efficiency of transfection was determined by RT-PCR from 2 µl of synthesized cDNA. PCR conditions were: 5 min incubation at 94°C followed by 28 cycles of 15 s at 94°C, 30 s at 60°C, and 30 s at 68°C, and a final incubation for 60 s at 68°C. The 18S ribosomal RNA gene was used as an internal expression control. The primers used were PR forward 5′-CCCGCCCTATCTCAACTACC-3′ and reverse 5′-GTTGTGCTGCCCTTCCATTTG-3’). 18S forward 5′-AGTGAAACTGCAATGGCTC-3′ and reverse 5′-TGACCGGGTTGGTTTTGAT-3’.

### Migration Assay

The wound-healing assay was performed to determine the cell migration of U251 cells. 2.5×10^5^ cells were plated in 6-well slides with DMEM high glucose supplemented until reaching 70% confluence. Then, cells were transfected as was described in the previous section. About 48 h after transfection and in 90% of confluence, a scratch was made using a 200 µl pipette tip. Floating cells were removed with PBS and DMEM medium (*In vitro*, S.A., CDMX., MEX) without phenol red and free of hormones, supplemented with 10% SFB also free of hormones were added again. Cytosine β-D-arabinofuranoside hydrochloride (10 µM, Ara-C, C1768, Sigma-Aldrich, St. Louis, MO, USA) was used to inhibit cell proliferation 1 h before adding the treatments. Cells were treated with P4 (50 nM), or vehicle (DMSO 0.001%). Four random fields were chosen per treatment to determine cell migration after 0, 6, and 12 h of treatment. Photographs were taken with an Infinity 1-2C camera (Lumenera, CA) connected to the inverted microscope Olympus CKX41 (Olympus, JPN).

### Co-Immunoprecipitation

Cell cultures were lysed in a buffer containing 50 mM Tris–HCl (pH 7.4), 150 mM NaCl, 1 mM EDTA, 1% Triton X-100, 0.1% SDS, and a cocktail of protease inhibitors (Sigma Aldrich, St. Louis, MO USA, # P8340) at 4°C overnight. Cell lysates were centrifuged at 12,500 rpm for 15 min. One mg of total protein present in the supernatant was incubated with 2 µg of antibody anti-PR (Santa Cruz Biotechnology Dallas, Texas, USA Ref B-30 sc-811) and 50 µL of sepharose-coupled protein A/G plus-agarose (sc-2003; Santa Cruz Biotechnology) under permanent agitation at 4°C overnight. The next day, samples were centrifuged, and the pellets washed three times with buffer (20 mM Tris HCl; 150 mM NaCl; 1mM EDTA, 0.1 Triton X-100, and a cocktail of protease inhibitors (Sigma Aldrich, St. Louis, MO USA, # P8340 pH 7.5). Finally, the samples were denatured by boiling in loading buffer (120 mM Tris, pH 6.8; 4% SDS; 0.2% glycerol; 5% β-mercaptoethanol; and 10 mg/ml bromophenol blue) and separated in SDS-PAGE. Western blot for cSrc was done as previously described in the Protein extraction and Western blotting section.

### TCGA Data Analysis

RNA-Seq counts from 196 grade II, 223 grade III, and 139 grade IV gliomas were obtained from Glioblastoma and Low-Grade Glioma projects of The Cancer Genome Atlas (TCGA) repository (https://portal.gdc.cancer.gov/). The data were downloaded and processed using TCGAbiolinks package version 2.12.6 for R.17 Additionally, expression profiles of 249 healthy brain cortex samples were obtained from the GTEx database (https://gtexportal.org/home/). Data were normalized by DESeq2 version 1.22.2 and plotted. Gene expression correlation in glioblastoma, from the TCGAbiolinks package for R.

### Statistical Analysis

**Figure 1:**
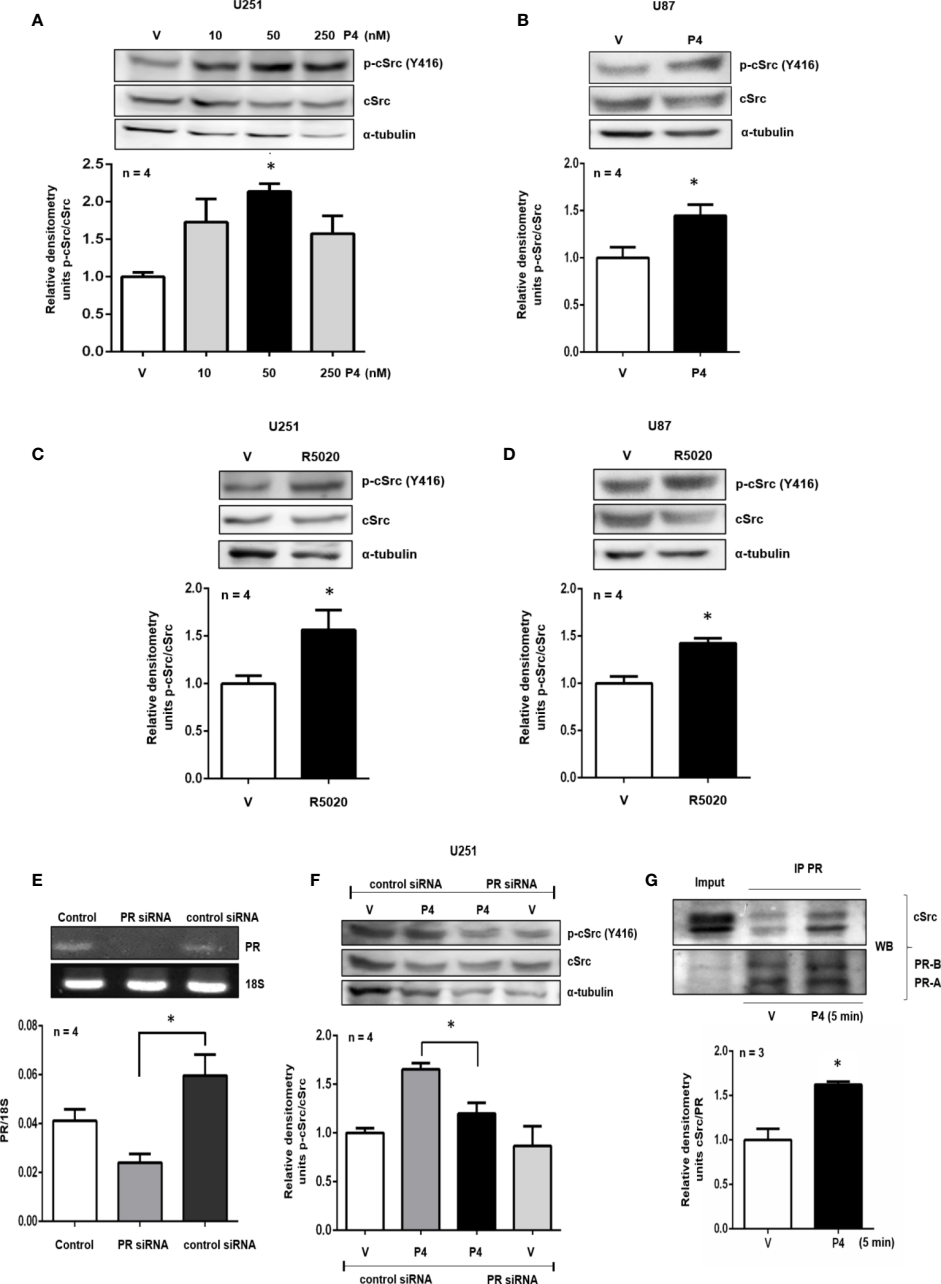
P4 induces the activation of cSrc through PR. **(A, B)** U251 and U87 cells were treated with P4 (10, 50 and 250 nM) and P4 (50 nM) respectively or vehicle (V, DMSO 0.01%) for 10 min. **(C, D)** U251 and U87 cells were treated with R5020 (10 nM) or vehicle (V, DMSO 0.01%) for 10 min. **(E)** U251 cells were transfected with PR siRNA and a control siRNA (an aleatory RNA sequence) (100 nM) or were only treated with lipofectamine (Control). **(F)** Transfected cells with PR siRNA or control siRNA were treated with P4 (50 nM) or vehicle (V, DMSO 0.01%) for 10 min. Upper panels show the representative western blots for p-cSrc, cSrc, and α-tubulin or representative RT-PCR bands for PR and 18S mRNA. Lower panels show the densitometric analysis. **(G)** U251 cells were treated with P4 (50 nM) or vehicle (V, DMSO 0.01%) for 5 min and co-immunoprecipitated with PR. Data were normalized respect to the vehicle or control. Results are expressed as the mean ± S.E.M. **(A–F)** n = 4 **(G)** n = 3; *p < 0.05.

**Figure 2:**
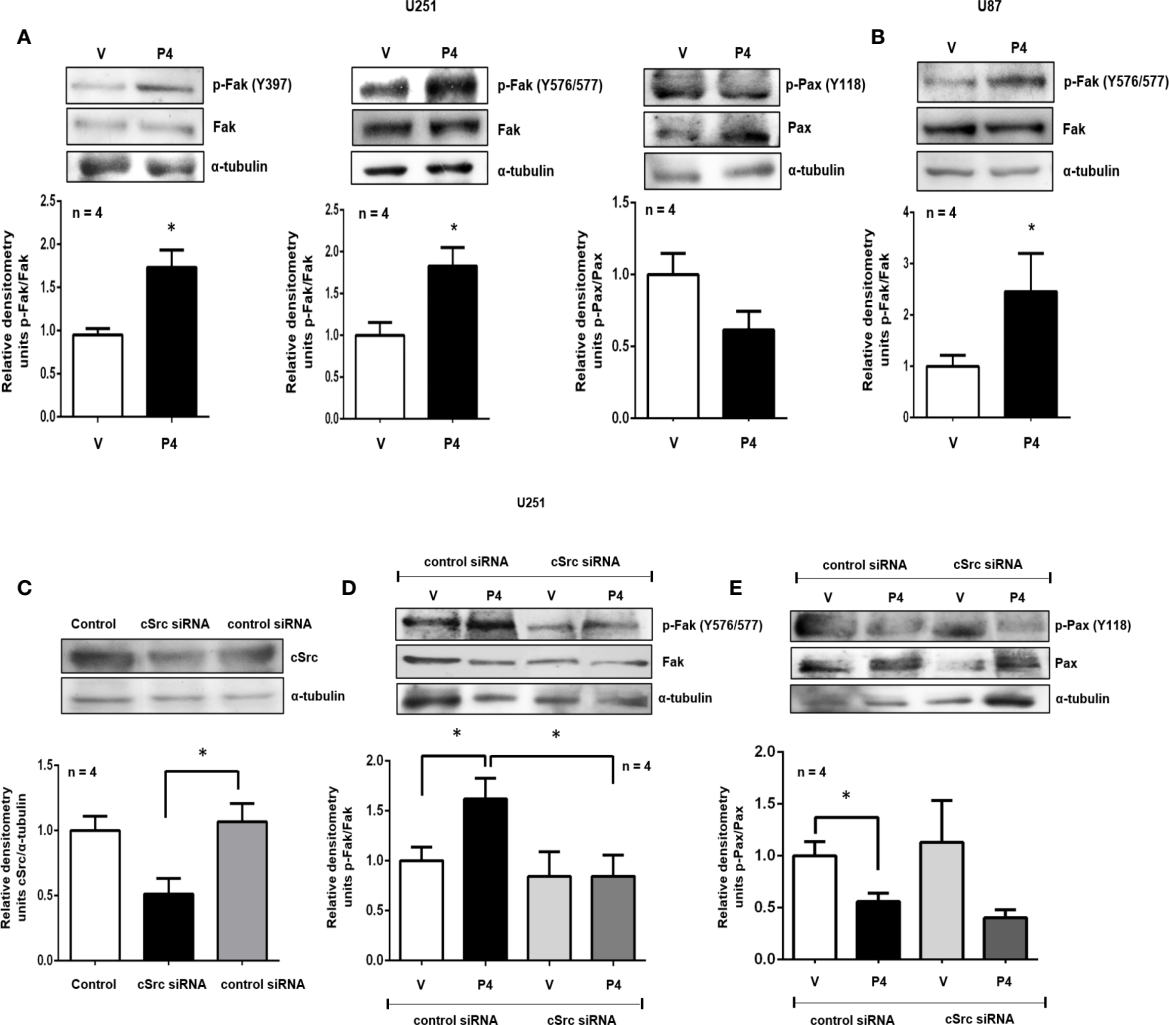
P4 induces the activation of Fak and Pax through cSrc in glioblastoma cells. **(A, B)** U251 and U87 cells were treated with P4 (50 nM) or vehicle (V, DMSO 0.01%) for 20 min. **(C)** U251 cells were transfected with cSrc siRNA and a control siRNA (an aleatory RNA sequence) (100 nM) or were only treated with lipofectamine (Control). **(D, E)** Transfected cells with cSrc siRNA or control siRNA were treated with P4 (50 nM) or vehicle (V, DMSO 0.01%) for 20 min. Upper panels show the representative western blots for, cSrc, p-Fak, Fak, p-Pax, Pax, and α-tubulin. Lower panels show the densitometric analysis. Data were normalized respect to the vehicle or control. Results are expressed as the mean ± S.E.M. n = 4; *p < 0.05.

**Figure 3:**
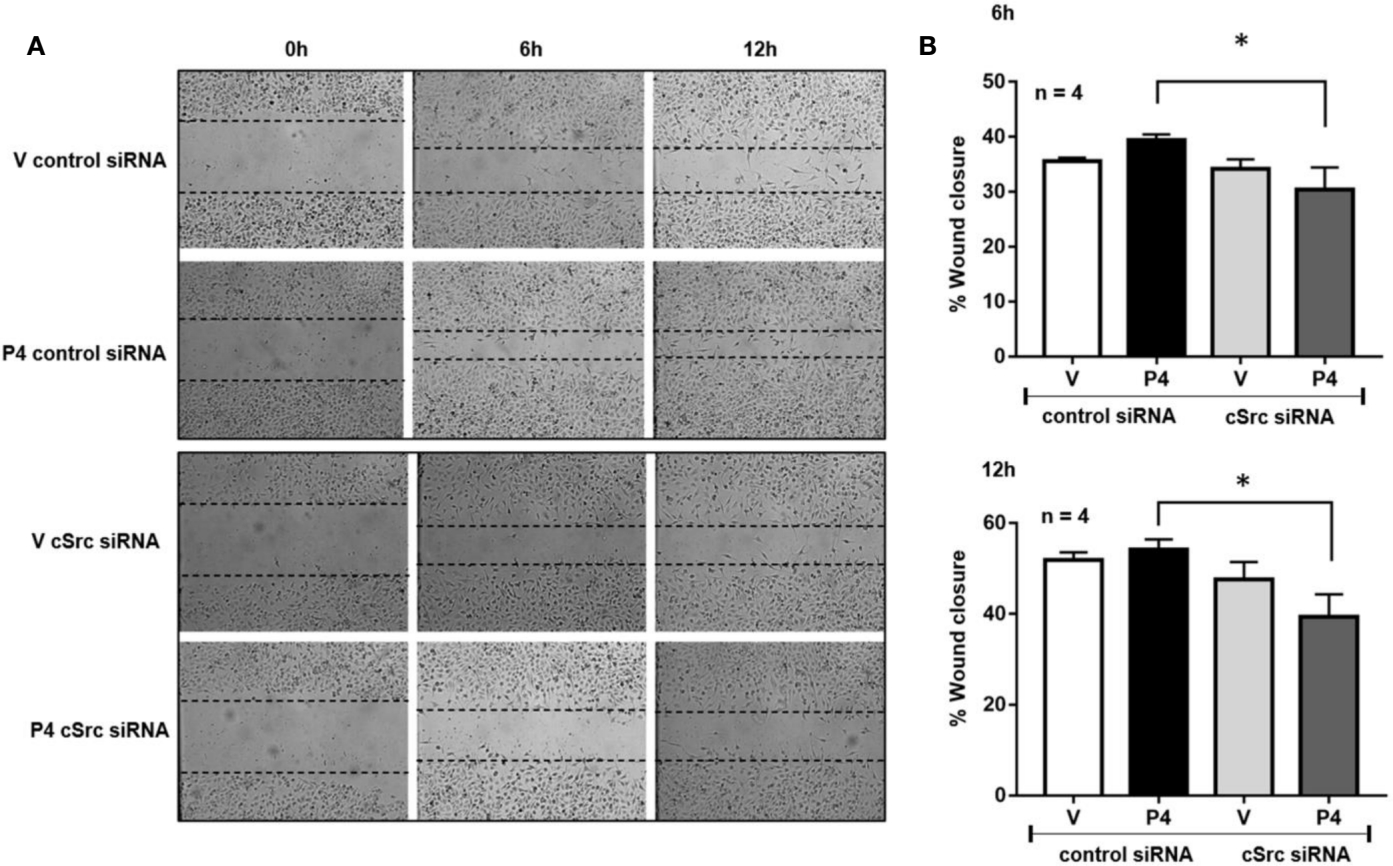
cSrc participates in cell migration induced by P4. U251 cells transfected with cSrc siRNA and a control siRNA (an aleatory RNA sequence) (100 nM) were treated with P4 (50 nM) or vehicle (V, DMSO 0.01%) in cells. Photographs from the scratch area were taken at 0, 6 and 12 h and were captured with 400× magnification. **(A)** Representative image of the scratch area. **(B)** Graph of the wound closure (%), determined in the scratch area. Results are expressed as the mean ± S.E.M. n = 4; *p < 0.05.

Data were and analyzed using Graph Pad Prism 5 program (GraphPad Software, Inc., USA). A one-way ANOVA with Bonferroni *post hoc* test (**Figures 1A, E, F**, **2C–E**, **3B**) or t-student test were used to establish the statistical differences between comparable groups. Values of p <0.05 were considered statistically significant.

## Results

### Activation of cSrc by P4 Is Mediated by PR in Glioblastoma Cells

**Figure 4:**
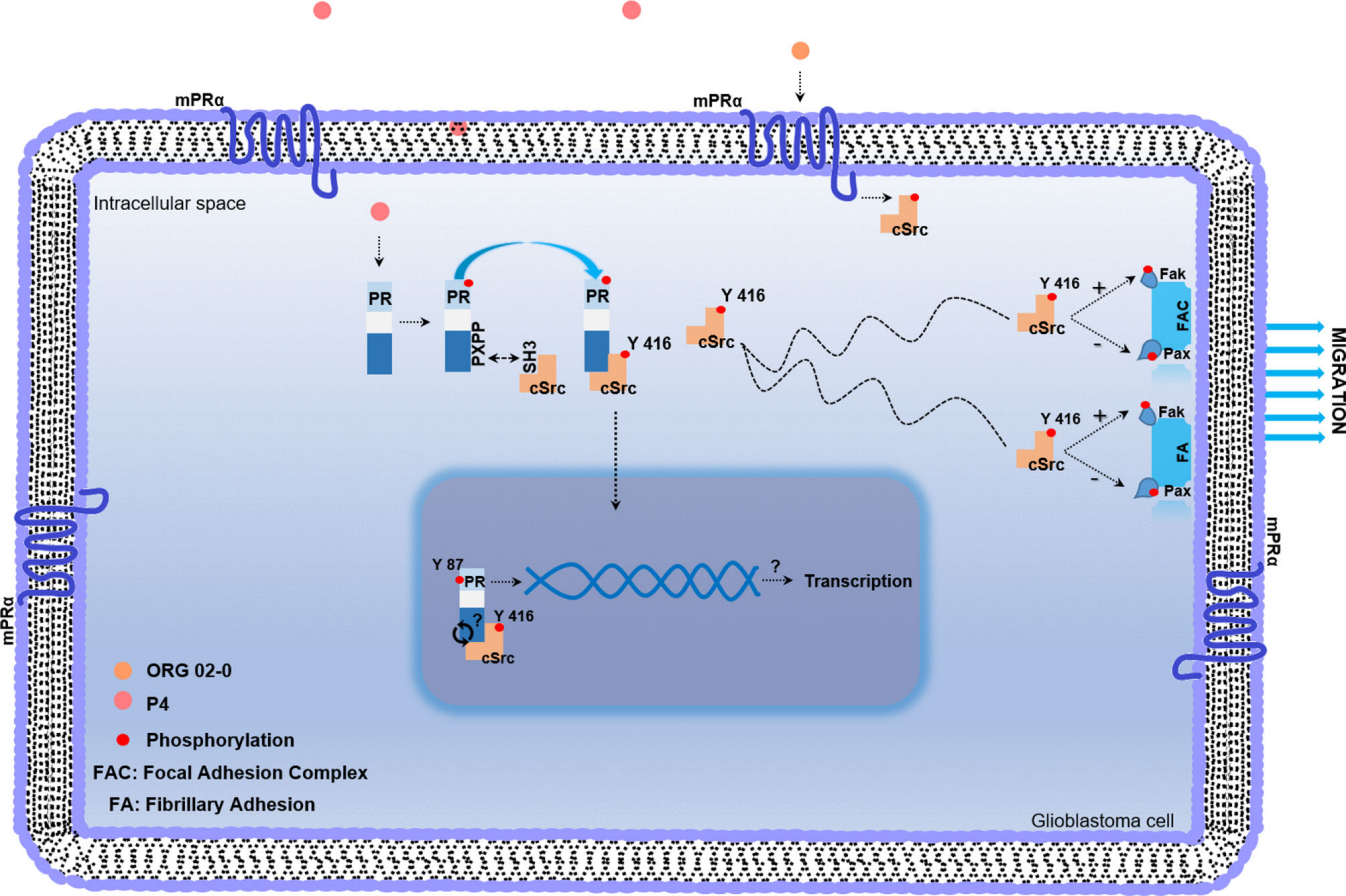
Working model of non-genomic PR mechanism of action in glioblastoma cell. Once P4 enters the cell, it induces phosphorylation of PR in serine and threonine residues. After activation, PR recruits cSrc through the interaction between the polyproline (PXPP) and the SH3 domains of PR and cSrc, respectively. This interaction induces a conformational change in cSrc, which exposes its catalytic domain, and promotes the autophosphorylation of cSrc. Once activated, cSrc participates in regulating the phosphorylation of focal adhesion and fibrillary adhesion components such as Fak and Pax, which finally lead to the migration process. mPRs are expressed in glioblastoma cells and the agonist of mPRα (ORG 02-0) induces the activation of cSrc. The putative role of cSrc in the tyrosine 87 phosphorylation of PR could be involved in the transcriptional activity of PR.

The role of PR and cSrc in breast cancer has been broadly studied. The stimulation of breast cancer cells with P4 activated cSrc through PR, and induced various signaling pathways that conducted to cancer progression (21–23). To test the potential role of P4 in cSrc activation in glioblastoma cells, at the beginning of the study, a time-dependence assay (0–60 min) using P4 (10 nM) was performed in U251 cells (**Supplementary Figure 1**), however, a significant effect on p-cSrc/cSrc ratio was not observed, and we decided to test higher P4 concentrations, at 10 (**Figures 1A, B** **)** and 15 min (**Supplementary Figure 2**). U251, and U87 cells were treated with three different concentrations of P4 (10, 50 and, 250 nM) for 10 min, and the phosphorylation of cSrc (Y416) was determined by western blot. P4 induced cSrc activation at 50 nM in U251 and U87 cells (**Figures 1A, B** **)**. P4 has affinity for other receptors besides PR (24, 25); Nevertheless, because of the high affinity of R5020 for PR (Kd ≈ 2 nM) (20) over other receptors (AR 1% binding affinity) (26–28), cells were also treated with 10 nM of R5020. As in the case of P4, R5020 increased the p-cSrc (Y416)/cSrc ratio in U251 and U87 cells (**Figures 1C, D** **)**. To finally demonstrate that cSrc activation by P4 was mediated by its intracellular PR, U251 cells were transfected with a commercial siRNA against PR or control siRNA (scramble sequence) and treated with P4 for 10 min at 50 nM. The efficiency of transfection was higher than 50% (**Figure 1E**). P4 induced the activation of cSrc in cells with control siRNA as in the previous experiments. In contrast, the siRNA against PR blocked the increase in p-cSrc (Y416)/cSrc ratio induced by P4 (**Figure 1F**). This result demonstrates the participation of PR in the cSrc activation by P4 (**Figure 4**). Considering the short time (10 min) for the activation of cSrc, this result suggests that PR exerts this effect through nongenomic actions.

### cSrc and PR Interact in U251 Cells

Non-genomic actions of PR are associated with the polyproline domain which can interact with SH3 domains of a variety of proteins including cSrc. In breast cancer cell lines, a physical interaction between cSrc and PR has been demonstrated (22). Co-immunoprecipitation assay was performed to evaluate the interaction between PR and cSrc. U251 cells were immunoprecipitated with antibodies against PR, and a western blot was carried out. In both vehicle and P4 treated cells, a band corresponding to cSrc was detected, indicating that PR (isoforms A and B) and cSrc directly or indirectly interact in glioblastoma cells. The treatment with P4 increased the interaction between PR and cSrc (**Figure 1G**). This result suggests that activation of cSrc is possible because of the physical interaction between cSrc and PR in glioblastoma cells (**Figure 4**).

### P4 Induces a Switch in Fak-Phosphofak and Pax-Phosphopax Ratios Through cSrc in Glioblastoma Cells

Fak and Pax are two of the most critical components of the focal adhesion complex, fundamental to regulating cell migration and invasion. To test if P4 was able to induce Fak and Pax activation, U251 and U87 cells were treated with P4 at 50 nM for 20 min, and their phosphorylation was determined by western blot. P4 (50 nM) increased the p-Fak/Fak ratio (Y397 and Y576/577) in U251 cells and the p-Fak/Fak ratio (Y576/577) in U87 cells, while in the case of Pax, P4 decreased the p-Pax/Pax ratio (Y118) in U251 cells at 20 min (**Figures 2A, B** **)**. cSrc is one of the major kinases implicated in the phosphorylation of focal adhesion complex components, especially Fak (29). To test the role of cSrc in the phosphorylation of Fak and Pax, U251 cells were transfected with a commercial siRNA against cSrc or control siRNA (scramble sequence) and treated with P4 (50 nM) for 20 min. The efficiency of transfection was 50% (**Figure 2C**). The siRNA against cSrc blocked the increase in the p-Fak/Fak (Y576/577) ratio induced by P4 (**Figure 2D**), but not the decrease in p-Pax/Pax ratio (**Figure 2E**). This result indicates that P4 is involved in regulating focal adhesion complex through PR and cSrc in glioblastoma cells (**Figure 4**).

### Silencing of cSrc Reduces the Migration Induced by P4

Previous results suggest that P4 and cSrc have a fundamental role in the migration of glioblastoma cells, which in turn, participate in the recurrence of this tumor (17, 30). To determine whether silencing of cSrc modify the migration induced by P4 in U251 cells, a scratch-wound assay was performed. In cells transfected with control siRNA and treated with P4, a slight increase in migration was observed as compared to vehicle at 6 and 12 h after treatment. This increase was inhibited in cells transfected with cSrc siRNA (**Figures 3A, B** **)**. This result demonstrates that the migration of glioblastoma cells induced by P4 is related to the activation of cSrc and reinforces the previous molecular findings.

### cSrc Has Several Putative Phosphorylation Sites Over PR

Phosphorylation of nuclear receptors, including PR, has great relevance in functions executed by these proteins. PR phosphorylation has been broadly studied in serine residues; however, there is scarce information about tyrosine residues. The potential role of active cSrc over PR phosphorylation was determined using three different databases (NetPhos 3.1, KinasePhos, and GPS 5.0). We found the same putative tyrosine residue in the amino acid 87 of PR in all of them. In the GPS 5.0 database, this residue presented the highest score, which means that it also has the highest potential for phosphorylation (**Table 1**). Even when this result must be confirmed in experimental assays, the information obtained by the databases opens the possibility of future investigation of functions and regulation of PR by cSrc phosphorylation (**Figure 4**).

**Table 1:** *In silico* analysis of putative phosphorylation sites of cSrc over PR.

	ID	Position	AA	Kinase	Score	E-value	Peptide
Netphos 3.1	Progesterone Receptor Homo sapiens AAA60081.1	87	Y	SRC	0.516		VEGAYSRAE
				EGFR	0.444		
KinasePhos		87	Y	SRC		13	VEGAYSRAE
GPS 5.0		87	Y	TK/SRC/SRCA/YES1	27.217		LSDVEGAYSRAEATR

Three different databases to predict phosphorylation sites were used to analyze the potential role of cSrc in PR phosphorylation. One putative tyrosine residue in the amino acid 87of PR was found in three databases: NetPhos 3.1, KinasePhos, and GPS 5.0.

### PXN (Pax) and PTPN12 (PTP-PEST) Expression Depends on Tumor Grade and Glioblastoma Subtype

PXN and PTPN12 expression data from 196 grade II, 223 grade III, and 139 grade IV (glioblastoma) astrocytomas were obtained from TCGA and compared to 249 healthy human brain cortex samples from the GTEx database. The expression of these genes was also compared among the four subtypes of glioblastomas defined by Verhaak and colleagues (31). The PXN and PTPN12 mRNA expression increased in glioblastomas compared to normal brain, and in the case of PTPN12, the expression was higher in glioblastomas compared to astrocytomas grades II and III (**Supplementary Figure 3A**). The analysis of expression among the four subtypes of glioblastomas showed that PXN and PTPN12 have the highest levels of expression in the mesenchymal subtype (the most aggressive glioblastoma subtype) (32) (**Supplementary Figure 3A**). The analysis of gene expression correlation between PXN and PTPN12 revealed a value of 0.61 (significant positive correlation) (**Supplementary Figure 3B**).

## Discussion

Glioblastoma is the most malignant brain tumor. Patients with glioblastoma have an overall survival of 14 months (1). One of the main influencing factors in the poor prognosis of these patients is the high capacity of glioblastoma cells to migrate and invade the brain parenchyma surrounding the tumor, which in turn makes extremely difficult a complete surgical resection (33). Several molecular signals are implicated in the processes of migration and invasion in glioblastoma; some are activated by cSrc kinase protein that belongs to the Src Family Kinase (SFK) (8, 34). Of all of the other family members (FYN, YES, BLK, YRK, FGR, HCK, LCK, and LYN) cSrc is the most often associated with cancer progression (35). This kinase has been associated with migration and invasion of multiple malignancies through the regulation of actomyosin contraction, actin polymerization (36), and ECM proteolysis (37). In glioblastomas, SFKs play an essential role in events related to motility and disruption of ECM.

It has been demonstrated that PR activated by P4 promotes the migration and invasion of glioblastoma cells (17). However, there is no information about the possible interplay between PR and cSrc in glioblastoma cells. In this work, we first investigated the capacity of P4 to activate cSrc through its PR and how this activation regulates the phosphorylation/dephosphorylation of kinases related to migration and invasion of glioblastoma cells. U251 and U87 cells were treated with P4 for 10 min, and the activation of cSrc was evaluated by western blot. The most effective concentration of P4 was 50 nM. The increase in the p-cSrc (Y416)/cSrc ratio in U251 cells was evident. Y416 is the amino acid residue localized in the domain SH1, which contains the autophosphorylation site required for the full cSrc activation (38). When U87 cells were treated with P4, a significant increase in the p-cSrc (Y416)/cSrc ratio was observed. Therefore, P4 induces the activation of cSrc in human glioblastoma derived cell lines.

In colorectal cancer, the increasing activity of cSrc rather than its overexpression is associated with metastasis (39, 40). cSrc is one of the first and most studied proto-oncogenes (41); however, its role in cancer progression is not entirely understood. The central role attributed to this kinase was increasing cellular proliferation (42). However, most recent investigations have found that cSrc regulates processes such as adhesion, invasion, and motility (38, 43). For example, the overexpression of cSrc in colon cancer does not induce the proliferation rate increase, but it facilitates the spread of cells (37). Some colleagues consider that cSrc induces cellular proliferation at the first stage of cancer development but regulates migration and invasion processes at the later stages (29). It has been demonstrated an essential role of SFKs in the motility of glioblastoma cells (30, 44). The activation of cSrc by P4 may be involved in the regulation of events associated with the migration and invasion of glioblastoma cells.

One of the first identified substrates of cSrc was Fak, a non-receptor tyrosine kinase closely related to regulating a variety of cellular processes, including cell migration (45). At the focal adhesion complexes, cSrc induces Fak’s phosphorylation and facilitates the turnover of these junctions, an essential step to cell migration. The complex Fak-cSrc can also phosphorylate Pax, which recruits other components to focal adhesion sites (38). It has been reported elevated levels of Fak expression in anaplastic astrocytoma and glioblastoma tumor biopsy samples compared to normal brain (46). In this work, we evaluated the capacity of P4 to activate Fak and Pax. P4 promoted the increase in the p-Fak(Y397)/Fak and p-Fak(Y576/577)/Fak ratio that corresponds to the autophosphorylation site and to another site directly phosphorylated by cSrc, respectively. Thus, P4 induces Fak’s phosphorylation, including in the tyrosine residues directly related to cSrc and with the turnover of focal adhesions (38). To determine if P4 induces the phosphorylation of Fak through cSrc, we transfected U251 cells with a commercial siRNA against cSrc or with control siRNA and treated them with P4 in the same conditions of the previous experiments. In this case, P4 failed to induce Fak activation in cells transfected with siRNA against cSrc.

Pax is a multifunctional protein that plays a scaffolding role at focal adhesions. Overexpression of this protein has been associated with high-grade astrocytomas as well with a poor survival (10). Upon integrin activation, Pax is mainly phosphorylated at two different tyrosine residues, namely Y31 and Y118, but this phosphorylation state is not permanent. Zaidel-Bar and colleagues found that tyrosine-phosphorylated Pax is associated with focal complex and focal adhesions, while non-phosphorylated Pax is associated with fibrillary adhesions. These colleagues proposed a hypothetical model in which Pax is initially phosphorylated and recruited to integrin adhesions. The rate of this recruitment is regulated by the presence of both phosphopax and Pax. Finally, phosphopax is dephosphorylated at a high rate under mechanical force, and the phosphorylation is reestablished at a low rate (47). In this work, we observed that P4 decreased the p-Pax(Y118)/Pax ratio in U251 cells 20 min after the treatment. This result suggests that P4 should induce Pax recruitment towards the integrins at fibrillary adhesions and contributes to the presence of both phosphorylated and unphosphorylated state, which is necessary to migration processes. When U251 cells were transfected with the siRNA against cSrc, the reduction in p-Pax(Y118)/Pax was more evident, which is in line with the role of cSrc in Pax phosphorylation. One of the proteins closely related to the dephosphorylation of Pax is the tyrosine phosphatase PTP-PEST. Shen and colleagues found that PTP-PEST coimmunoprecipitates with Fak and Pax in chicken embryo cells (48). These colleagues also demonstrated that the expression of PTP-PEST decreases the phosphotyrosine on Pax (49). In glioblastoma, PTP-PEST regulates the invasion events by phosphorylation-dependent ubiquitination of essential focal proteins such as Cas, Fak, Pax, and Src (11). Bioinformatic analysis revealed that PXN and PTPN12 mRNA expression was higher in astrocytomas (Grades II, III, and IV) compared to normal brain and showed the highest expression in the mesenchymal subtype (the most aggressive glioblastoma subtype, associated with bad prognostic) (32). The analysis of gene expression correlation revealed a value of 0.61 (significant positive correlation). These results suggest that these proteins together are implicated in the progression of glioblastomas. In the same line, the scratch-wound assay analysis showed that silencing of cSrc in U251 cells abolished the increase in cell migration induced by P4. Interestingly in 2013, Matias-Sanchez and colleagues found that PR, stimulated by P4 and the synthetic progestin medroxyprogesterone acetate, have an essential role in the actin polymerization, branching, and focal adhesion complex formation in cortical neurons. The molecular mechanism proposed by these colleagues involucrate the activation of Fak, and other proteins related to migration, such as WAVE and moesin. Phosphorylation of the latter was promoted by PR through the Ras homolog gene family, member A and Rho-associated kinase-2. Therefore, we should not underestimate the role of these last proteins in P4 effects (50).

The observed effects induced by P4 could also be mediated by membrane progesterone receptors (mPRs), G protein-coupled receptors that belong to the Progestin and AdipoQ Receptor Family (PAQR). Five subtypes of mPRs (mPRα, mPRβ, mPRδ, mPRϵ, and mPRγ) have been identified, and they are expressed in human glioblastoma cells (51, 52). Importantly, the activation of mPRα by ORG 02-0, a specific mPRα agonist, induces proliferation, migration, and invasion through the activation of cSrc and Akt in human derived glioblastoma cells (24).

P4 can exert its effects through various receptors in glioblastoma cells (24, 25). Therefore, an agonist of PR (R5020) was used to treat the U251 and U87 cells, and in both cases, an increase in p-cSrc (Y416)/cSrc ratio was observed. Considering the high affinity of R5020 for the PR and that this progestin is unable to be transformed into the active metabolites of P4 (53), it makes sense to think that results previously described are a consequence of the action of P4 through its PR and not by another receptor or metabolite. To determine if cSrc activation by P4 was induced through PR, a more specific assay was conducted. U251 cells were transfected with a commercial siRNA against PR or with control siRNA and treated with P4 in the same conditions of the previous experiments. As is shown in **Figure 1F**, P4 failed to induce cSrc activation in cells transfected with siRNA against PR. Thereby, the effect of P4 over cSrc activation is mediated by PR in these human glioblastoma cells.

Even though PR is widely known for its role as a transcription factor (53, 54), in the last two decades the attention has been focused on the actions that it can exert out of the nucleus (55). Non-genomics actions of PR are due to a polyproline domain (amino acids 396–456) that can interact with the SH3 domain of several proteins, including cSrc. Once this interaction occurs, the intramolecular interactions that hold cSrc in a closed configuration are disrupted, and the autophosphorylation site is exposed (22). This mechanism has been broadly studied in breast cancer. It has been demonstrated that in breast cancer cells, P4 can promote the interaction between PR and cSrc and, in turn, inducing proliferation (56), migration, and invasion (19). To test the interaction between PR and cSrc we performed a co-immunoprecipitation assay. This assay shows that PR (isoforms A and B) and cSrc interact in glioblastoma cells and that P4 enhances this interaction. This result suggests that activation of cSrc by P4 is due to a conformational change in cSrc that enables the autocatalytic domain to be exposed. However, this result must be interpreted with care since some colleagues have found that in breast cancer cell lines, the activation of cSrc through the PR is dependent on the estrogen receptor (ER). ERα plays an essential role in breast cancer cells by activating the Src/Erk pathway and increasing cell proliferation. Estrogens or progestins can induce this effect; however, according to studies conducted by Migliaccio and colleagues and Ballaré and colleagues, it is necessary to form a complex including ERα, PR, and Src (21, 23). Boonyaratanakornkit and colleagues, on the contrary, support the idea of PR self-sufficiency to induce cSrc activation without ER. They found that in breast cancer cells no expressing ER, P4 induced the activation of cSrc through PR (22). In glioblastomas, estradiol increased cell growth, migration, invasion, and the epithelial–mesenchymal transition (EMT) through activation of ERα (57, 58); therefore, we cannot dismiss the idea of the role of ERα in the PR-cSrc interaction.

Phosphorylation of PR is a post-translational modification broadly studied. Among other functions, it is directly related to regulating the transcriptional activity of this receptor and the degradation by the proteasome (59, 60). Serine and threonine phosphorylation of PR has been widely investigated (60–62); however, there are no reports about PR tyrosine phosphorylation. Therefore, we performed an *in silico* analysis in three databases to search putative phosphorylation sites of cSrc over PR. NetPhos 3.1, KinasePhos, and GPS 5.0. predicted the putative tyrosine residue in the amino acid 87 with the highest score. Even when *in vitro* and *in vivo* experiments are mandatory to demonstrate this prediction, we can speculate about this post-translational modification’s possible role. The phosphorylation of the estrogen receptor by SFKs proteins has been studied in breast cancer cell lines. Its inhibition reduces its stability and transcriptional activity and alters the ligand binding (63).

Then, the interaction of PR and cSrc in glioblastoma cells could be bilateral and involve the genomic and non-genomic actions of P4. In conclusion, this work is the first report in demonstrating the interaction between cSrc and PR in human glioblastoma cells. This interaction induces cSrc activation, which in turn participates in the regulation of the activity of proteins involved in the migration and invasion of glioblastomas. The results presented here open new perspectives for the treatment of glioblastomas.

## Data Availability Statement

Publicly available datasets were analyzed in this study. This data can be found here: https://portal.gdc.cancer.gov/

## Author Contributions

All authors contributed to the study conception and design. Material preparation, data collection, and analysis were performed by CB-A and AD-M. The first draft of the manuscript was written by CB-A and AD-M and reviewed by IC-A. AG-A participated in the analysis of results, and all authors commented the versions of the manuscript. All authors contributed to the article and approved the submitted version.

## Funding

This work was financially supported by DGAPA-PAPIIT (IN217120), UNAM, Mexico and by a scholarship to CB-A from Consejo Nacional de Ciencia y Tecnología (277679), Mexico.

## Conflict of Interest

The authors declare that the research was conducted in the absence of any commercial or financial relationships that could be construed as a potential conflict of interest.

## Abbreviations

CNS, central nervous system; Fak, Focal adhesion kinase; Pax, paxillin; ECM, extracellular matrix; PTP-PEST, (tyrosine-protein phosphatase non-receptor type 12); TCGA, The Cancer Genome Atlas; P4, progesterone; PR, progesterone receptor; SFK, Src Family Kinase; ER, estrogen receptor, mPRs, membrane progesterone receptors; PAQR, Progestin and AdipoQ Receptor.
