# Supplementary material for: Intracellular Progesterone Receptor and cSrc Protein Working Together to Regulate the Activity of Proteins Involved in Migration and Invasion of Human Glioblastoma Cells
Source: Front Endocrinol (Lausanne). 2021 Mar 26;12:640298. doi: 10.3389/fendo.2021.640298 (PMC8032993; doi:10.3389/fendo.2021.640298)
Supplement: Supplementary file 1 [file DataSheet_1.docx]

Supplementary Material

Intracellular Progesterone Receptor and cSrc Protein Working Together to Regulate the Activity of Proteins Involved in Migration and Invasion of Human Glioblastoma Cells

**Claudia Bello-Alvarez^1^,** **Aylin Del Moral-Morales^1^, Aliesha González-Arenas^2^ and Ignacio Camacho-Arroyo^1^***

**Correspondence:** Dr. Ignacio Camacho-Arroyo: [camachoarroyo@gmail.com](mailto:camachoarroyo@gmail.com)

# Supplementary Figures.


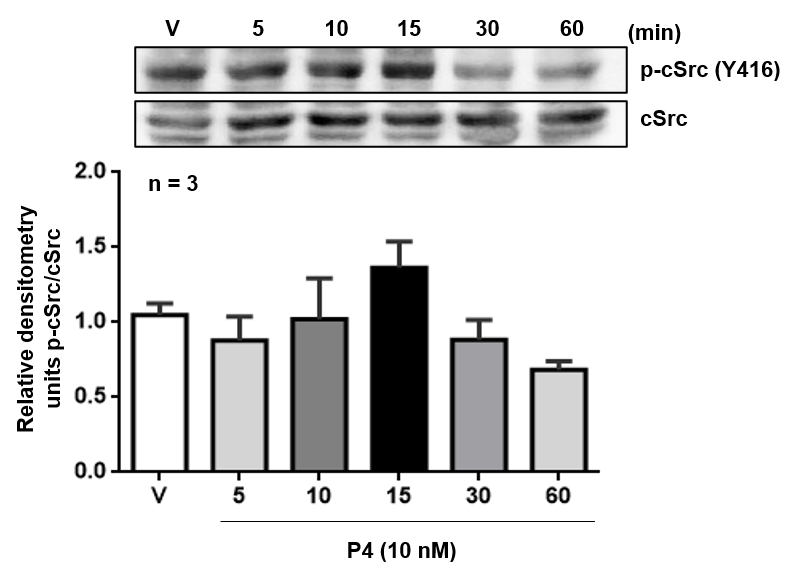


**Supplementary Figure 1. Effect of P4 on cSrc activation in glioblastoma cells at different periods of time.** U251 cells were treated with P4 (10 nM) or vehicle (V, DMSO 0.01%) for 5, 10, 15, 30 and 60 min. Upper panel shows a representative western blot for p-cSrc and cSrc. Lower panel shows the densitometric analysis. Data were normalized respect to the vehicle. Results are expressed as the mean ± S.E.M. n= 3.


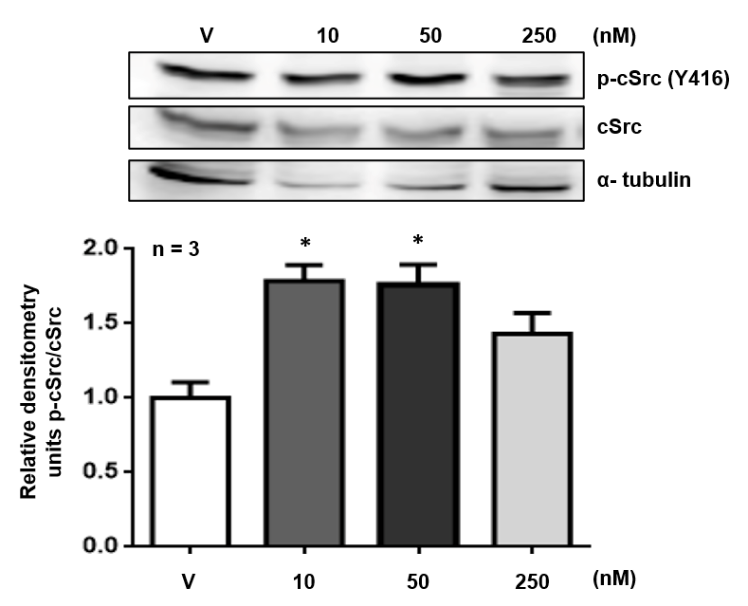


**Supplementary Figure 2. Effect of different P4 concentrations on cSrc activation in glioblastoma cells.** U251 cells were treated with P4 (10, 50 and 250 nM) or vehicle (V, DMSO 0.01%) for 15 min. Upper panel shows a representative western blot for p-cSrc and cSrc. Lower panel shows the densitometric analysis. Data were normalized respect to the vehicle. Results are expressed as the mean ± S.E.M. n= 3 *p<0.05 vs V.


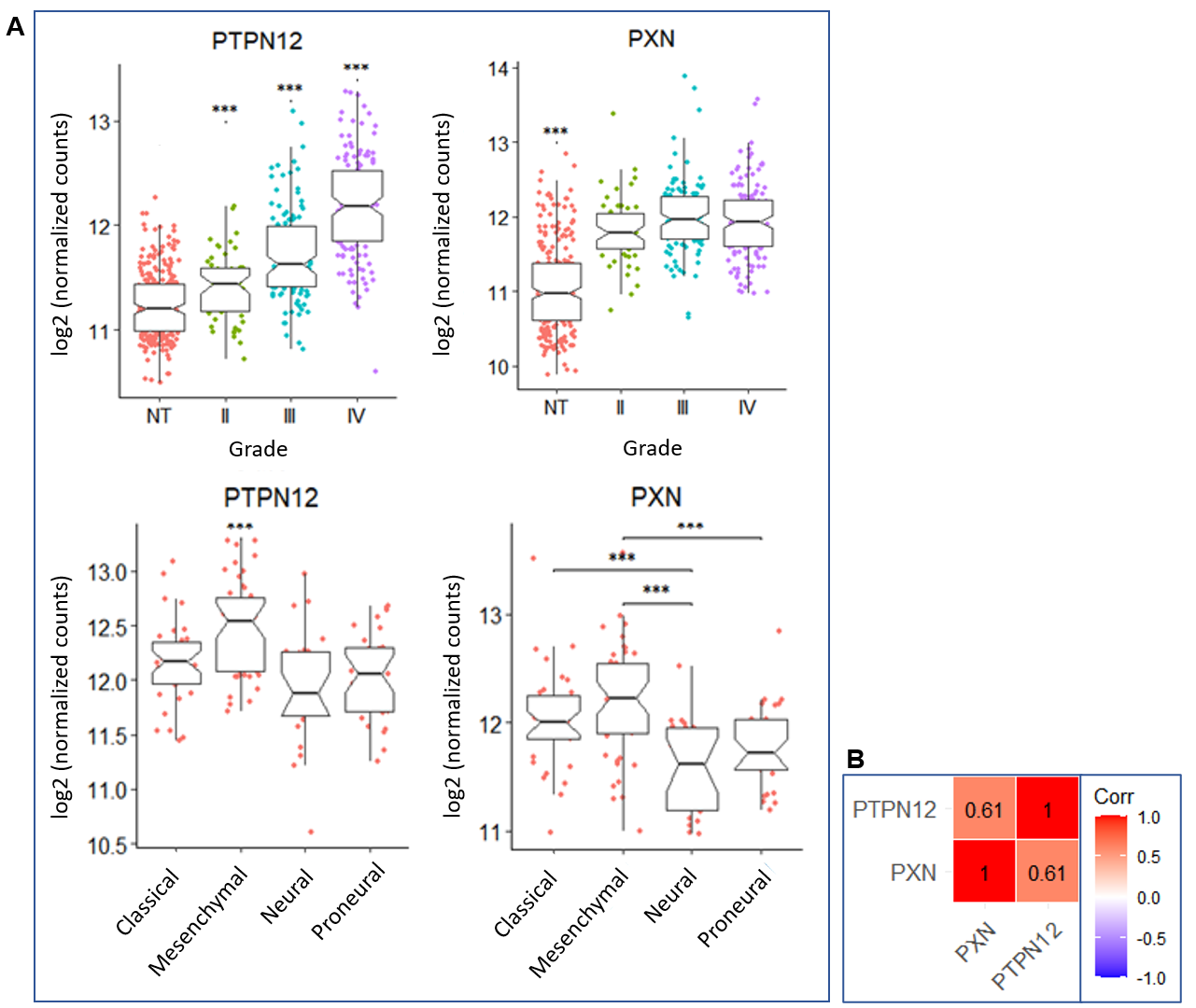


**Supplementary Figure 3. PTPN12 (PTP-PEST) and PXN (Pax) expression depends on tumor grade and glioblastoma subtype.** (A) Upper graphs: PTPN12 (PTP-PEST) and PXN (Pax) expression in human astrocytoma primary tumor biopsies. RNA-Seq data from 322 astrocytoma primary tumors (196 grade II, 223 grade III, 139 grade IV or glioblastoma (GBM) were obtained from TCGA repository and compared against 249 samples of normal brain cortex obtained from GTEx database. PTPN12***p<0.001 all grades vs NT, grade IV vs III, and grade III vs II; PXN ***p<0.001 NT vs all grades. Lower graphs: PTPN12 (PTP-PEST) and PXN (Pax) expression in glioblastoma subtypes. RNA-Seq counts from GBM subtypes: classical (CL), mesenchymal (MES), neural (N), and proneural (PN) obtained from TCGA. PTPN12 *p<0.05. mesenchymal vs all others, and PXN ***p<0.001. mesenchymal vs neural, mesenchymal vs proneural. (B) Gene expression correlation between PTPN12 and PXN in glioblastoma, from the TCGAbiolinks package for R. Significant positive correlation (0.61).
